# Fasting‐Induced Hepatic Gluconeogenesis Is Compromised In *Anxa6* ^*−/−*^ Mice

**DOI:** 10.1002/jcp.70084

**Published:** 2025-08-13

**Authors:** Anna Alvarez‐Guaita, Marc Bernaus‐Esqué, Patricia Blanco‐Muñoz, Yangjing Liu, David Sebastian, Elsa Meneses‐Salas, Mai K. Linh Nguyen, Antonio Zorzano, Francesc Tebar, Carlos Enrich, Thomas Grewal, Carles Rentero

**Affiliations:** ^1^ Unit of Cell Biology, Department of Biomedical Sciences, Faculty of Medicine and Health Sciences University of Barcelona Spain; ^2^ Fundació de Recerca Clínic Barcelona—Institut d'Investigacions Biomèdiques August Pi i Sunyer (FRCB‐IDIBAPS) Barcelona Spain; ^3^ Departament de Bioquímica i Fisiologia, Facultat de Farmàcia i Ciències de l'Alimentació Universitat de Barcelona Spain; ^4^ Institute of Biomedicine of the University of Barcelona (IBUB) Barcelona Spain; ^5^ Centro de Investigación Biomédica en Red de Diabetes y Enfermedades Metabólicas Asociadas (CIBERDEM), Instituto de Salud Carlos III Madrid Spain; ^6^ School of Pharmacy, Faculty of Medicine and Health University of Sydney NSW Australia; ^7^ Institute for Research in Biomedicine (IRB Barcelona) The Barcelona Institute of Science and Technology Barcelona Spain; ^8^ Departament de Bioquímica i Biomedicina Molecular, Facultat de Biologia Universitat de Barcelona Spain

**Keywords:** alanine, alanine‐dependent gluconeogenesis, Annexin A6, hypoglycaemia, SNAT

## Abstract

Maintaining constant blood glucose levels is essential for energizing glucose‐dependent tissues. During the fed state, insulin lowers elevated blood glucose, while in the fasted state, glucagon maintains blood glucose levels through hepatic stimulation of fatty acid oxidation, glycogenolysis, and gluconeogenesis (GNG). The liver plays a crucial role in these metabolic adaptations. Deregulation of GNG is a hallmark of type 2 diabetes mellitus (T2DM), driven by hepatic insulin resistance, elevated glucagon levels, and excess circulating free fatty acids. The glucose metabolism of 8‐ to 12‐week‐old WT and *Anxa6* knock‐out (*Anxa6* ^*−/−*^) mice was analysed during regular feeding and fasting using indirect calorimetry, tolerance tests and biochemical analysis. Despite normal insulin‐sensitive control of glucose levels and effective glycogen mobilization, *Anxa6* ^*−/−*^ mice display rapid hypoglycaemia during fasting. This metabolic disarrangement, in particular during the early stages of fasting is characterized by a low respiratory exchange ratio (RER) and increased lipid oxidation during the diurnal period, indicating a reliance on lipid oxidation due to hypoglycaemia. Elevated glucagon levels during fasting suggest deficiencies in GNG. Further analysis reveals that *Anxa6* ^*−/−*^ mice are unable to utilize alanine for hepatic GNG, highlighting a specific impairment in the glucose‐alanine cycle in fasted *Anxa6* ^*−/−*^ mice, underscoring the critical role of ANXA6 in maintaining glucose homeostasis under metabolic stress. During fasting, slightly reduced expression levels of alanine aminotransferase 2 (*Gpt2*) and lactate dehydrogenase (*Ldha2*), enzymes converting alanine to pyruvate, and the hepatic alanine transporter SNAT4 might contribute to these observations in the *Anxa6* ^*−/−*^ mice. These findings identify that ANXA6 deficiency causes an inability to maintain glycolytic metabolism under fasting conditions due to impaired alanine‐dependent GNG.

**Abbreviations**
ALAT: alanine aminotransferase
ANXA6: Annexin A6
AUC: area under the curve
BCAAs: branched‐chain amino acids
BOH: beta‐hydroxybutyrate
CV: coefficient of variation
FBP: fructose‐1,6‐bisphosphatase
G6Pase: glucose‐6‐phosphatase
GAP: GTPase activating protein
GEO: Gene Expression Omnibus
GNG: gluconeogenesis
HFD: high‐fat diet
i.p.: intraperitoneal
LDH: lactate dehydrogenase
NAFL: nonalcoholic fatty liver disease
NASH: nonalcoholic steatohepatitis
PEPCK: phosphoenolpyruvate carboxykinase
RER: respiratory exchange ratio
SNAT: sodium‐coupled neutral amino acid transporter
T2DM: type 2 diabetes mellitus
WT: wild type

## 1 Introduction

Circulating blood glucose levels need to be steadily maintained irrespective of dietary nutritional inputs or during fasting periods to supply glucose‐dependent tissues (central nervous system, red blood cells and renal medulla). During the fed state, high levels of glucose induce insulin secretion, which stimulates glucose uptake by cells, and activates anabolic pathways such as glycogen production and lipogenesis and inhibits catabolic pathways. In the fasted state, blood glucose levels are maintained by elevated glucagon levels stimulating hepatic release of glucose generated by fatty acid oxidation, glycogenolysis and gluconeogenesis (GNG) (Lin and Accili 2011). The liver plays an essential role in this metabolic adaptation through housing processes such as lipogenesis, glycogen production, fatty acid oxidation, glycogenolysis and GNG to control glucose homeostasis.

During metabolic stress such as fasting, the liver is responsible for maintaining blood glucose levels (Rui 2014). Initially, elevation of blood glucagon levels triggers glucose production and secretion from hepatic glycogenolysis (glycogen breakdown). Once glycogen stores are depleted, glucose is then *de novo* synthetized from various gluconeogenic precursors, including amino acids from extrahepatic tissues. When in excess, dietary amino acids even stimulate hepatic GNG in the fed state (Just et al. 2020; Shulman and Landau 1992). Importantly, deregulation of GNG is a hallmark of type 2 diabetes mellitus (T2DM), and greatly contributes to highly elevated blood glucose levels in diabetic patients. In the latter, GNG is increased due to insulin resistance failing to inhibit hepatic glucose production, elevated glucagon levels activating pro‐GNG signalling pathways, and excess of circulating free fatty acids driving GNG (Jiang et al. 2020).

Supply for primary carbon skeletons used for GNG may derive from pyruvate, lactate, glycerol and amino acids. During fasting, glutamine and alanine account for 60%–80% of amino acids released from skeletal muscle (Young 1991), with alanine being the main hepatic GNG substrate via the Cahill cycle (Cahill 2006; Sarabhai and Roden 2019). In hepatocytes, alanine is mainly taken up by the system A ubiquitously expressed Na^+^‐coupled neutral amino acid transporter (SNAT) 2 and the liver‐specific SNAT4 (Mackenzie and Erickson 2004). Little is known about the in vivo regulation of these SNAT transporters, although feedback mechanisms for SNAT4 expression and sinusoidal plasma membrane localization during liver development and regeneration have been reported (Alvarez‐Guaita et al. 2020; Kondou et al. 2013). Interestingly, the retromer complex, which orchestrates endocytic recycling and maintains cell surface abundance of nutrient transporters (Gallon and Cullen 2015), delivers SNAT2 to the plasma membrane upon amino acid withdrawal to prevent its degradation in lysosomes (Curnock et al. 2019). Importantly, the GTPase RAB7 regulates the recruitment of several retromer subunits to endosomes and cargo recognition (Rojas et al. 2008). This process is inhibited by the RAB7‐GTPase activating protein (GAP) TBC1D5 (Seaman et al. 2009), which promotes RAB7 inactivation and enables fusion of autophagosomes to lysosomes (Gutierrez et al. 2004). As retromer expression is elevated upon amino acid starvation, this indicates that transcriptional control of endosomal recycling provides an important means for the adaptive regulation of nutrient uptake (Curnock et al. 2019).

Annexin A6 (ANXA6) is a calcium‐dependent phospholipid‐binding protein highly abundant in the liver that is involved in membrane trafficking (Enrich et al. 2011), epidermal growth factor receptor and RAS/mitogen‐activated protein kinase signalling (Enrich et al. 2011; Grewal et al. 2010), plasma‐membrane microdomain organisation (Alvarez‐Guaita et al. 2015), and cholesterol homeostasis through the regulation of RAB7 activity (García‐Melero et al. 2016; Meneses‐Salas et al. 2020; Reverter et al. 2011). Specifically, ANXA6 recruits the RAB7‐GAP TBC1D15 to late endosomes, promoting RAB7 inactivation (Meneses‐Salas et al. 2020). *Anxa6* knockout (*Anxa6* ^*−/−*^) mice appear normal, with slight alterations in cardiomyocyte function (Song et al. 2002) and adiponectin secretion (Krautbauer et al. 2017). Yet, when assessed under challenging physiological conditions, such as high‐fat diet, ANXA6 deficiency identified an inability to inhibit insulin‐dependent GNG (Cairns et al. 2018). Moreover, our recent work revealed ANXA6 to critically control the survival of mice during liver regeneration (Alvarez‐Guaita et al. 2020). During this process, the remnant liver needs to maintain hepatic functions that control blood glucose homeostasis. However, during the hepatic regeneration program, *Anxa6* ^*−/−*^ mice were unable to produce glucose *de novo* due to SNAT4 mislocalization, which severely impaired hepatic alanine uptake, the main gluconeogenic substrate during the regeneration process (Alvarez‐Guaita et al. 2020).

Little is known about the function of ANXA6 in the regulation of glucose homeostasis under physiological stress conditions such as fasting. Here, we demonstrate that *Anxa6* ^*−/−*^ mice showed no differences in blood glucose secretion or absorption capability, yet these mice were unable to maintain blood glucose levels in the initial stages of fasting and during the fasting state. While glycogen mobilization and GNG from pyruvate and glutamine were not affected, *Anxa6* ^*−/−*^ mice were unable to produce glucose *de novo* from alanine. After 24 h of fasting, the liver of *Anxa6* ^*−/−*^ mice displayed slightly reduced expression levels of alanine aminotransferase 2 (*Gpt2*), lactate dehydrogenase (*Ldha2*), and the alanine transporter SNAT4 (*Slc38a4*). Altogether our findings identify an unexplored defect upon ANXA6 deficiency related to the feedback control mechanisms that link nutrient sensing with metabolic enzyme expression and acid transporter recycling in the liver of *Anxa6* ^*−/−*^ mice.

## 2 Methods

### 2.1 Animals

Eight‐ to twelve‐week‐old C57Bl6/J wild type (WT) and whole‐body *Anxa6* ^*−/−*^ male mice (Hawkins et al. 1999) were used for all experiments and were maintained in a 12 h light/dark cycle, allowed food (regular low‐cholesterol, low‐fat cereale based rodent chow diet (2014 Teklad Global 14% protein rodent maintenance diet; Envigo)) and water ad libitum. Every effort was made to minimize animal suffering and to use the minimum number of animals per group and experiment. All the animal care and experimental procedures were approved by the Local Ethical Committee of the University of Barcelona following European (2010/63/UE) and Spanish (RD 53/2013) regulations for the care and use of laboratory animals.

### 2.2 Indirect Calorimetry

Mice energy expenditure was measured by open circuit indirect calorimetry using the Oxymax 8‐chamber system (Columbus Instruments). Before recording the rates of oxygen consumption (VO_2_) and carbon dioxide production (VCO_2_), mice were allowed to adapt to the standard Oxymax chambers for 2 days. VO_2_ and VCO_2_ measured at 22°C for 24 h, for 1.5 min in 20‐min intervals for each animal. RER, glucose and lipid oxidation were calculated according to the following equations: RER = VCO_2_/VO_2_; Glucose oxidation [g/min] = 4.55 ∗ VCO_2_ − 3.21 ∗ VO_2_; and Lipid oxidation [g/min] = 1.67 ∗ VO_2_ − 1.67 ∗ VCO_2_ (Frayn 1983). Mice activity (ambulation) was monitored as events of each mice traversing the cage (counts/hour).

### 2.3 Glucose Metabolism In Vivo Studies

For glucose and insulin tolerance tests, mice were fasted for 5 h before intraperitoneal (i.p.) injection of 2 g/kg glucose or 0.75 U/kg insulin. For the determination of GNG, mice were fasted for 24 h before i.p. injection of 2 g/kg sodium pyruvate, glycerol, lactate, l‐glutamine or l‐alanine. Blood glucose levels were determined from blood obtained by a small incision in the mouse tail using a glucometer (Glucocard G+ meter set, Arkray) at 0, 15, 30, 60, and 120 min after injection.

### 2.4 Glycogen Quantification

To assess liver glycogen content, a 200 mg liver sample was homogenized in 1 ml 30% KOH at 100°C for 10 min. Following the hydrolysis, the samples were allowed to cool to room temperature, and 2 ml of ethanol was added. The mixture was then incubated for 24 h at −20°C to facilitate glycogen precipitation. After incubation, samples were centrifuged at 2,000 × g for 15 min at 4°C, and the supernatant was discarded. The resulting pellet was resuspended in 3 ml of 1:2 (v/v) mixture of distilled water and ethanol at 4°C, and the suspension was subjected to a second centrifugation at 2,000 × g for 15 min at 4°C. The pellet was then resuspended in 1 ml 5 N H_2_SO_4_ and incubated at 100°C for 2 h to ensure complete hydrolysis of the glycogen to glucose. Finally, the samples were neutralized with 1 N NaOH using phenolphthalein (Fluka) as pH indicator. Glucose concentrations, representative of glycogen levels, were quantified using the Glucose assay kit (Sigma) according to manufacturer's instructions.

### 2.5 Insulin and Glucagon Quantification

Insulin (US Mouse Insulin ELISA, Mercodia) and glucagon (Glucagon EIA kit, Sigma) plasma levels were determined undiluted by ELISA according to manufacturer's instructions. The insulin ELISA kit showed a coefficient of variation (CV) % between 2% and 9%, and the glucagon EIA kit showed a CV% between 1% and 16%.

### 2.6 Beta‐Hydroxybutyrate (BOH, Ketone Body) Quantification

For the determination of blood ketone bodies, blood was collected by intracardiac puncture in BD Blood Collection Microtainer tubes (BD PST^TM^ Lithium heparin/gel). Five µl of plasma sample was analysed with Ketone bodies kit (Sigma) following the manufacturer's instructions.

### 2.7 RNA Extraction and Quantitative Real‐Time PCR

Total RNA was prepared from mice liver using RNeasy Lipid Tissue Mini Kit (Qiagen) in accordance with the manufacturer's protocol. 1 µg RNA from each sample was reverse‐transcribed using High Capacity cDNA Reverse Transcription Kit (Applied Bioscience). In a final volume of 20 µl real‐time PCR Brilliant SYBRGreen QPCR Master Mix (Agilent Technologies), 10 µl of 1:20 diluted cDNA as a template and specific primers (see Table 1) together with a standard PCR amplification protocol (10 min at 95°C, 45 cycles of 30 s at 95°C, 15 s at 60°C and 30 s at 72°C, 10 s at 95°C and 60 s at 65°C) and the LightCycler system (Roche Diagnostics) were used for real‐time PCR reaction according to manufacturer's instructions. Relative gene expression data was analysed following the 2^−ΔΔCt^ method (Livak and Schmittgen 2001).

**Table 1:** Mouse specific primer sequences for real‐time PCR.

Gene symbol (protein)	Forward primer (5′ – > 3′)	Reverse primer (5′ – > 3′)
*Pck1* (PEPCK)	GTCTGGCTAAGGAGGAAGGG	GCCAGGAGCAATCCAAAAA
*G6pc2* (G6Pase)	CACGCCTTTTGCTGGACTCG	AGGGGGATGGACGCACTTTTACA
*Fbp1* (FBP1)	ACCTGCCTGCACCTTTAGTC	TTGGTTGAGCCAGCGATACC
*Gpt1* (ALAT1)	TCCAGGCTTCAAGGAATGGAC	CAAGGCACGTTGCACGATG
*Gpt2* (ALAT2)	CAGACCCAGACAACATTTACCTG	CGCGGAGTACAAGGGATACTG
*Ldha* (LDH)	AGTTGTTGGGGTTGGTGCTGTTGG	GGGCCCCCGCGGTGATAAT
*Slc38a2* (SNAT2)	TGAAAAGCCATTATGCCGACG	CCCACAATCGCATTGCTCAG
*Slc38a4* (SNAT4)	CAGAAAGGCGGGAAAGGGCT	TGTTCATGGCGTCCTTGTCG
*Tbp* (housekeeping gene)	CACCCCTTGTACCCTTCAC	TTCACTCTTGGCTCCTGTGC

### 2.8 Preparation of Liver Lysates

All procedures were conducted at 4°C. Livers were removed from mice and liver tissue samples were placed in Lysing Matrix tubes (MP Biomedicals) with homogenization buffer (10 mM Tris, 150 mM NaCl, 5 mM EDTA, pH 7.5) containing 10 µg/ml of leupeptin and aprotinin, and 1 mM of orthovanadate, NaF and PMSF. Samples were then homogenized in a FastPrep120 homogenizer (MP Biomedicals) and stored at −20°C.

### 2.9 Plasma and Liver Amino Acid Levels

Plasma amino acid levels were quantified from 100 µl of plasma by cation‐exchange chromatography followed by post‐column derivatization with ninhydrin and UV detection as described (Moore et al. 1958). Proteins were precipitated with 100 µl 10% TCA. NorLeucine served as internal control.

### 2.10 ALAT Activity Quantification

For ALAT activity determination, a 200 mg liver sample was homogenized in 1.5 ml of 1 M Tris‐HCl, 150 mM NaCl and 5 mM EDTA, and filtered with a 0.45 µm PVDF filter. ALAT activity was then analysed using an autoanalyzer Advia 1650 (Bayer HealthCalre) according to manufacturer's instructions.

### 2.11 Western blot Analysis

Liver or cell lysates boiled in 1x sample buffer, resolved on SDS‐PAGE and transferred to Immobilon‐P (Millipore) membranes. Membranes were blocked in 5% nonfat milk, incubated overnight in primary antibodies, washed in TBST, incubated with HRP‐conjugated secondary antibodies (see Table 2) and developed using enhanced chemiluminescence ECL (NZYtech) and the density of each spot pixel on the membrane was determined by Image Quant LASS 4000 (GE Healthcare). ImageJ software was used for quantitative analysis of WB bands (Schneider et al. 2012).

**Table 2:** Specific antibodies used in this study.

Antibody	Company	Application
anti‐SNAT4 (rabbit polyclonal)	Our laboratory (Alvarez‐Guaita, 2020)	WB (1:1,000)
anti‐GAPDH (goat monoclonal)	Genescript Cat. No.A00191	WB (1:10,000)
anti‐ANXA6 (rabbit polyclonal)	Our laboratory (Garcia‐Melero, 2016)	WB (1:3,000)
anti‐beta ACTIN (mice monoclonal)	Sigma Cat. No.A5441	WB (1:5,000)
anti‐rabbit IgG‐HRP (secondary antibody)	Bio‐Rad Cat. No.170‐6515	WB (1:3,000)
anti‐goat IgG‐HRP (secondary antibody)	Promega Cat. No.V8051	WB (1:3,000)

### 2.12 Oil Red O Staining

Livers were taken from mice following anaesthesia (ketamine/xylazine) after intracardially perfusion with formaldehyde. Liver samples were then fixed with formaldehyde for 24 h and cryopreserved with 30% sucrose for 24 h. The samples were embedded in optimal cutting temperature (OCT) compound (Tissue‐Tek) at –80°C. Liver sections (7 µm thick) were then stained for Oil Red O and counterstained with haematoxylin.

### 2.13 Statistics

Data are shown as means ± SEM. Statistical comparison of two groups was performed using unpaired *t* test; analysis of interaction was performed with a two‐way ANOVA with ad hoc Bonferroni posttest. Symbols represent ∗*p* < 0.05, ∗∗*p* < 0.01, ∗∗∗*p* < 0.001. Statistical analysis was performed in GraphPad Prism 10.

## 3 Results

### 3.1 ANXA6 Deficiency Induces Metabolic Energetic Disarrangement in Mice

The liver, together with other organs, coordinates the systemic adaptation to metabolic fluctuations of glucose, fatty acids and amino acid levels. In previous studies we showed that ANXA6 plays an important role in the regulation of lipid and glucose metabolism under high‐fat diet (Cairns et al. 2018) or during liver regeneration (Alvarez‐Guaita et al. 2020). However, the potential function of ANXA6 in response to limited dietary nutrient availability remains to be elucidated. To explore this, we analysed publicly available metabolism‐related data sets deposited in the Gene Expression Omnibus (GEO) to assess *Anxa6* mRNA expression under various physiological and pathophysiological conditions. In a data set related to nonalcoholic fatty liver disease (GSE135251) (Govaere et al. 2020), *Anxa6* mRNA levels were significantly elevated in both nonalcoholic fatty liver (NAFL) and nonalcoholic steatohepatitis (NASH) liver of patients across different disease stages (Figure S1A). We also analysed hepatic *Anxa6* mRNA expression in mouse models subjected to 24 h fasting, high‐fat diet (HFD), and with genetic background of obesity (*ob/ob* mice) (GSE85439) (Yang et al. 2016). *Anxa6* mRNA liver expression increased after 24 h of fasting and returned to basal levels following 4 h of refeeding (Figure S1B). Similarly, hepatic expression was elevated after 48 h of acute HFD exposure but normalized following chronic HFD treatment for 12 weeks (Figure S1C). Additionally, *ob/ob* mice exhibited a statistically significant increase in hepatic *Anxa6* mRNA levels (Figure S1D). These findings suggest that *Anxa6* expression is dynamically regulated and may have functional relevance in both physiological and pathophysiological metabolic states.

**Figure 1:**
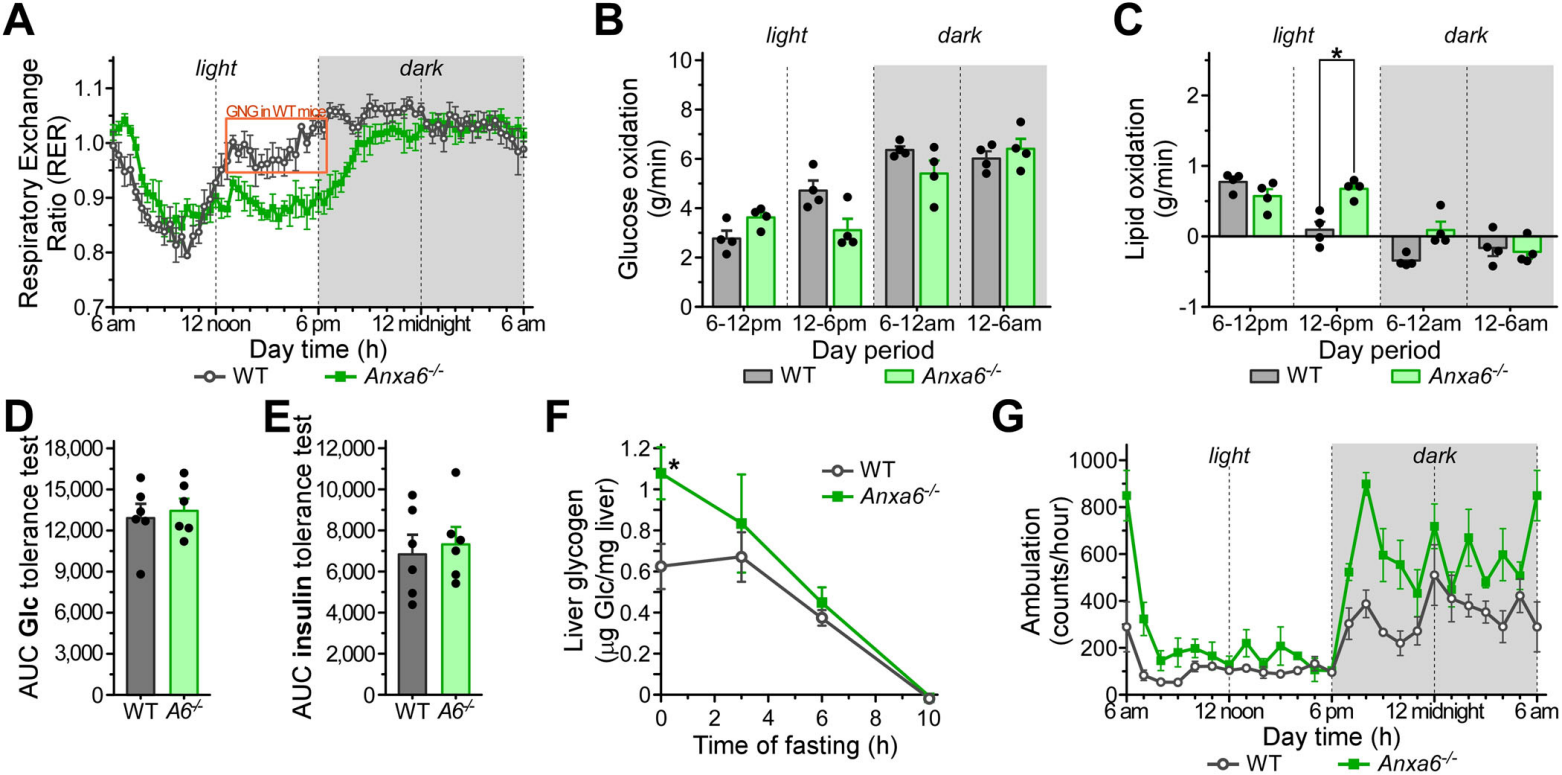
Energetic imbalance during voluntary fasting (light period) in *Anxa6* ^*−/−*^ mice. (A) Respiratory exchange ratio (RER) from WT and *Anxa6* ^*−/−*^ mice measured every 20 min (n = 4 mice per group). (B) Glucose oxidation flux expressed as the mean of 6‐h period during day and night‐time of WT and *Anxa6* ^*−/−*^ mice (n = 4 mice per group). (C) Lipid oxidation flux expressed as the mean of 6 h period during day and night‐time of WT and *Anxa6* ^*−/−*^ mice (n = 4 mice per group). (D) AUC from glucose tolerance test of WT and *Anxa6* ^*−/−*^ mice (n = 6 mice per group) after 5 h fasting administrating i.p. 2 g/kg glucose. (E) AUC from insulin tolerance test of WT and *Anxa6* ^*−/−*^ mice (n = 6 mice per group) administrating i.p. 0.75 U/kg insulin. (F) Liver glycogen levels in WT and *Anxa6* ^*−/−*^ mice (n = 3–6 mice per group and time point) during fasting. (G) Mice ambulation per hour from WT and *Anxa6* ^*−/−*^ mice (n = 4 mice per group). Data are expressed as means ± SEM. Data was analysed by two‐way ANOVA with Bonferroni's post‐hoc test (B, F) or unpaired *t* test (D, E), *p < 0.05, **p < 0.01, ***p < 0.001 comparing *Anxa6* ^*−/−*^ to WT mice.

Therefore, we initially assessed the general ability of *Anxa6* ^*−/−*^ mice to adapt to physiological changes that require hormone‐regulated maintenance of blood glucose levels. ANXA6 liver expression levels in WT and *Anxa6* ^*−/−*^ mice are shown in Figure S2A. We analysed the metabolic response in a normal light‐dark cycle, with voluntary and cyclical feeding and fasting periods. WT and *Anxa6* ^*−/−*^ mice were scrutinised in metabolic chambers for 24 h, and oxygen and carbon dioxide levels were monitored every 20 min. The respiratory exchange ratio (RER), an indicator of fuel utilization, along with glucose and lipid oxidation rates, were assessed (Figure 1A–C, Figure S2B‐D). *Anxa6* ^*−/−*^ mice exhibited consistently low RER values throughout the diurnal (inactive) period (6 am‐6 pm), suggesting a reliance on lipid oxidation during voluntary fasting. This was supported by markedly reduced glucose oxidation and significantly elevated lipid oxidation in *Anxa6* ^*−/−*^ mice compared to WT controls (Figure 1B‐C), indicating enhanced lipid‐based energy metabolism in the absence of ANXA6. In contrast, WT mice showed RER values near 1 during the late diurnal and nocturnal (active) phases, reflecting predominant carbohydrate utilization (Figure 1A). During the early light phase (6 am–12 noon), WT mice displayed lower RER values and decreased glucose oxidation (Figure 1A), consistent with increased fat oxidation before gluconeogenesis activation later in the day (12 noon–6 pm) (red box in Figure 1A, and Figure 1B‐C).

In line with our previous studies (Cairns et al. 2018), the insulin‐sensitive control of systemic glucose levels, glucose absorption and secretion were not negatively affected in the *Anxa6* ^*−/−*^ mice during regular feeding conditions (Figure 1D‐E). We next analysed the metabolism of glycogen, the initially mobilized hepatic source of glucose during early stages of hypoglycaemia (Figure 1F). As described earlier (Alvarez‐Guaita et al. 2020), the amount of hepatic glycogen stored in fed animals was significantly higher in ANXA6‐deficient mice. During early fasting (0–3 h), *Anxa6* ^*−/−*^ mice rapidly mobilized hepatic glycogen stores, indicating a premature induction of glycogenolysis. In contrast, WT mice showed a delayed response, with the initial glycogen mobilization occurring approximately 3 h after fasting onset and eventually reaching mobilization rates comparable to those of *Anxa6* ^*−/−*^ mice (Figure 1F). When body weight, food and water intake, urine volume and defecation were analysed in WT and *Anxa6* ^*−/−*^ mice (Figure S3), a statistically significant increase in *Anxa6* ^*−/−*^ mice water intake and urine volume was detected. Interestingly, *Anxa6* ^*−/−*^ mice showed higher activity both during the light and dark periods, which was statistically significant during the initial phase of these periods (Figure 1G, Figure S2E).

Hence, these data suggest energetic alterations of glucose metabolism in *Anxa6* ^*−/−*^ mice during the diary light period (voluntary fasting period), which correlates with the recently described GNG impairment during liver regeneration (Alvarez‐Guaita et al. 2020). Yet, ANXA6‐deficient mice presented a regular insulin and glucose response and metabolization, although showed higher glycogen stores in the liver.

### 3.2 Glucose Metabolism during Fasting in *Anxa6* ^*−/−*^ Mice

**Figure 2:**
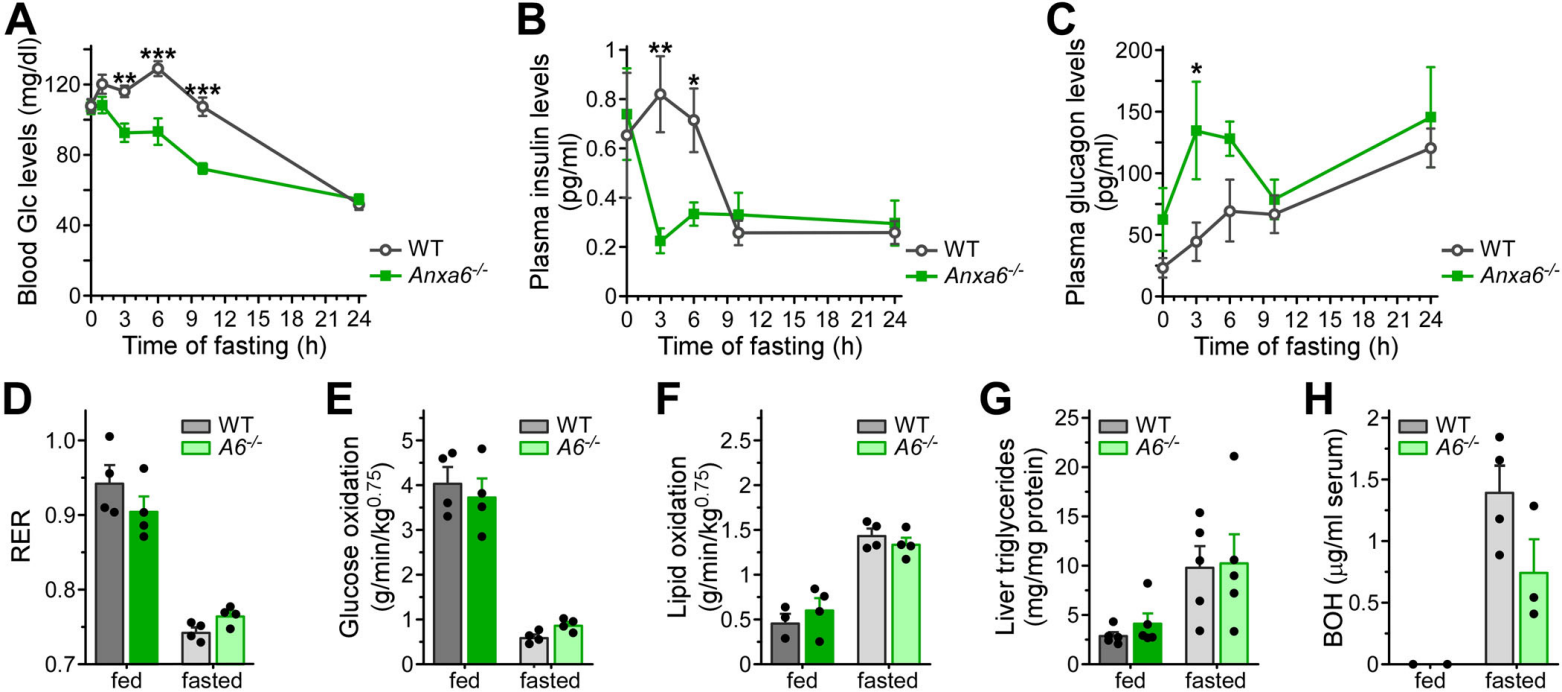
Glucose metabolism during fasting in *Anxa6* ^*−/−*^ mice. (A) Blood glucose levels in WT and *Anxa6* ^*−/−*^ mice (n = 13 mice per group) during fasting. (B) Plasma insulin levels in WT and *Anxa6* ^*−/−*^ mice (n = 6–9 mice per group) during fasting. (C) Plasma glucagon levels in WT and *Anxa6* ^*−/−*^ mice (n = 3–6 mice per group) during fasting. (D) Respiratory exchange ratio (RER) expressed as the mean value of a fed period and 12 h fasting period of WT and *Anxa6* ^*−/−*^ mice (n = 4 mice per group). (E) Glucose oxidation flux expressed as the mean value of a fed period and 12 h fasting period of WT and *Anxa6* ^*−/−*^ mice (n = 4 mice per group). (F) Lipid oxidation flux expressed as the mean value of a fed period and 12 h fasting period of WT and *Anxa6* ^*−/−*^ mice (n = 4 mice per group). (G) Hepatic triglyceride levels of WT and *Anxa6* ^*−/−*^ mice before and after 12 h fasting (n = 5 mice per group). (H) Blood β‐hydroxybutyrate (BOH, ketone body) levels of WT and *Anxa6* ^*−/−*^ mice before and after 12 h fasting (n = 4 mice per group). Data are expressed as means ± SEM. Data was analysed by two‐way ANOVA with Bonferroni's post‐hoc test, *p < 0.05, **p < 0.01, ***p < 0.001 comparing *Anxa6* ^*−/−*^ to WT mice.

To further characterize the energetic metabolism during fasting, WT and *Anxa6* ^*−/−*^ mice were deprived of food and the concentration of circulating blood glucose was measured over 24 h. Interestingly, and in contrast to the relatively constant glucose levels observed in WT mice during the first 10 h of fasting, *Anxa6* ^*−/−*^ mice exhibited a rapid and pronounced drop in blood glucose levels as early as the initial fasting time points (Figure 2A). In line with this lower blood glucose levels, plasma insulin levels declined rapidly and were significantly lower in *Anxa6* ^*−/−*^ mice when compared with their WT counterparts (Figure 2B). On the other hand, plasma glucagon levels were significantly higher during the early fasting period in *Anxa6* ^*−/−*^ mice compared to controls (Figure 2C). In spite of this, RER, glucose and lipid oxidation after 12 h fasting yielded nonsignificant differences between both mice strains (Figure 2D‐F). Similarly, triglyceride content did not change in *Anxa6* ^*−/−*^ mice after 12 fasting compared to WT (Figure 2G). Also, when lipid oxidation was measured by means of serum ketone bodies (β‐hydroxybutyrate), a slight but not significant reduction of serum ketone bodies was evident in *Anxa6* ^*−/−*^ mice after 12 h of fasting (Figure 2H). Moreover, Oil Red O staining displayed similar levels of lipid droplets both in WT and *Anxa6* ^*−/−*^ mice liver after 18 h of fasting (Figure S2F).

Altogether, these results demonstrate that *Anxa6* ^*−/−*^ mice were unable to maintain blood glucose levels under fasting conditions, manifested as significant hypoglycaemia immediately after food deprivation. This hypoglycaemia was produced even when glycogen stores were mobilized in the liver to secrete glucose. Interestingly, the consequent energy deficiency was alleviated using alternative energy sources such as lipid oxidation. This pointed at ANXA6 deficiency to trigger stress‐induced hypoglycaemia due to deficiencies in the utilization of alternative sources for glucose production, such as GNG. In line with this hypothesis, glucagon levels were highly elevated after 0–6 h of fasting in *Anxa6* ^*−/−*^ mice (Figure 2C), supporting an increased ability to rapidly degrade glycogen and suggesting deficiencies in GNG.

### 3.3 ANXA6 Is Essential for Alanine‐Dependent Hepatic Gluconeogenesis

**Figure 3:**
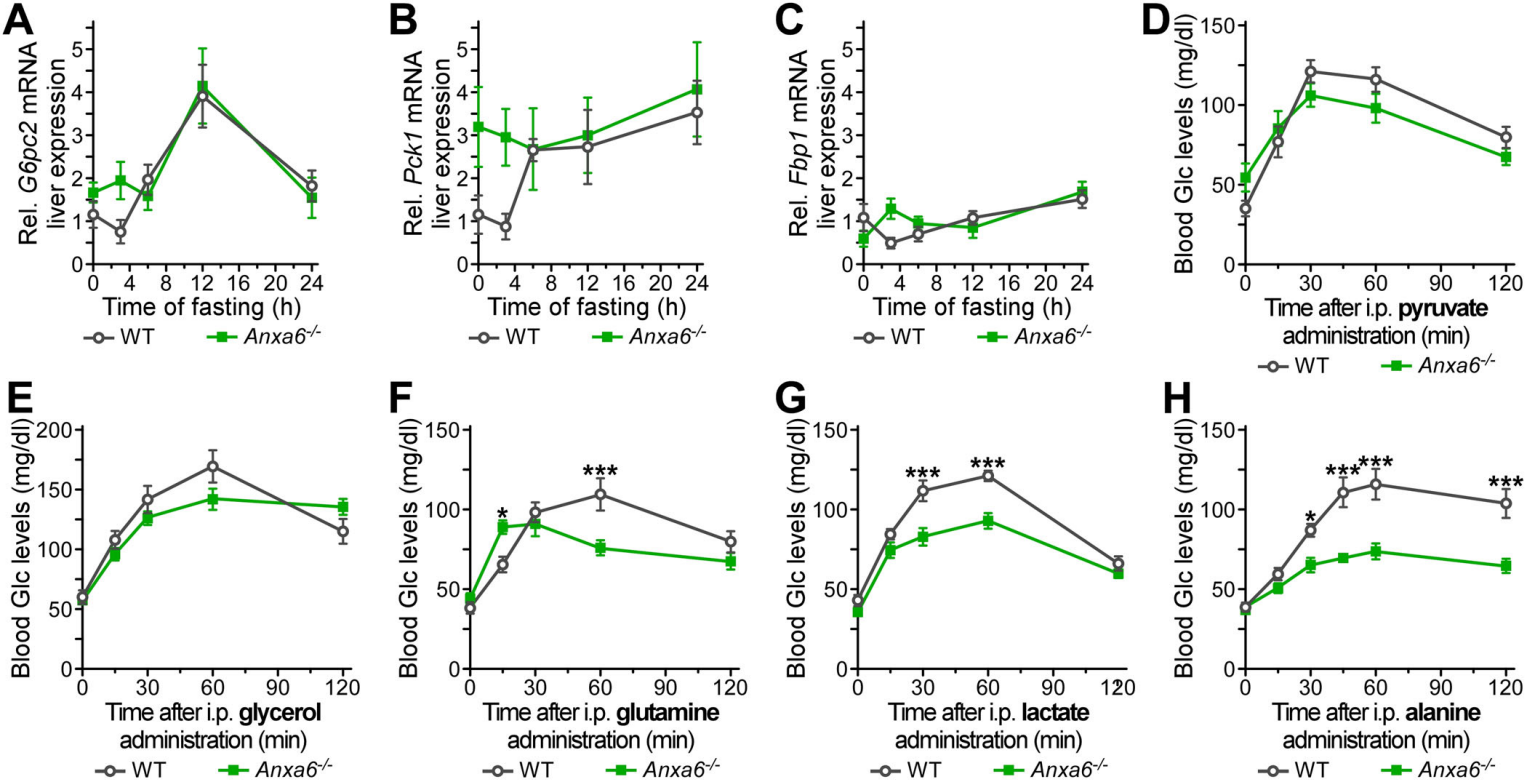
Hepatic gluconeogenic impairment in *Anxa6* ^*−/−*^ mice. (A) Relative liver mRNA expression levels of glucose‐6‐phosphatase (*G6pc2*) during fasting in WT and *Anxa6* ^*−/−*^ mice liver (n = 5 mice per group and time point). (B) Relative liver mRNA expression levels of phosphoenolpyruvate carboxykinase (*Pck1*) during fasting in WT and *Anxa6* ^*−/−*^ mice liver (n = 5 mice per group and time point). (C) Relative liver mRNA expression levels of fructose‐1,6‐bisphosphatase (*Fbp1*) during fasting in WT and *Anxa6* ^*−/−*^ mice liver (n = 5 mice per group and time point). (D) Pyruvate tolerance test of WT and *Anxa6* ^*−/−*^ mice (n = 6 mice per group) after 24 h fasting administrating i.p. 2 g/kg of sodium pyruvate. (E) Glycerol tolerance test of WT and *Anxa6* ^*−/−*^ mice (n = 6 mice per group) after 24 h fasting administrating i.p. 2 g/kg of glycerol. (F) Glutamine tolerance test of WT and *Anxa6* ^*−/−*^ mice (n = 11 mice per group) after 24 h fasting administrating i.p. 2 g/kg of l‐glutamine. (G) Lactate tolerance test of WT and *Anxa6* ^*−/−*^ mice (n = 11 mice pre group) after 24 h fasting administrating i.p. 2 g/kg of lactate. (H) Alanine tolerance test of WT and *Anxa6* ^*−/−*^ mice (n = 12 mice pre group) after 24 h fasting administrating i.p. 2 g/kg of l‐alanine. Data are expressed as means ± SEM. Data was analysed by two‐way ANOVA with Bonferroni's post‐hoc test, *p < 0.05, **p < 0.01, ***p < 0.001 comparing *Anxa6* ^*−/−*^ to WT mice.

Besides the induction of glycogenolysis in response to prolonged hypoglycaemia, the liver also upregulates *de novo* glucose production from the utilization of non‐carbohydrate carbon substrates such as pyruvate, glycerol, glutamine, and alanine. We therefore first analysed the expression of glucagon‐inducible key GNG genes such as phosphoenolpyruvate carboxykinase (PEPCK, *Pck1*), glucose‐6‐phosphatase (*G6pc2*), and fructose‐1,6‐bisphosphatase (*Fbp1*) (Wallace and Barritt 2005) in WT and *Anxa6* ^*−/−*^ during the 24 h fasting period. These data showed a trend, although not significant, towards upregulation of these genes (0–6 h), implicating elevated GNG during these initial stages of fasting in *Anxa6* ^*−/−*^ mice (Figure 3A‐C). To analyse whether hepatic GNG was altered in the absence of ANXA6, we next quantified circulating glucose levels during in vivo tolerance tests providing pyruvate, glycerol, lactate, glutamine or alanine as substrates. No significant differences were observed in WT and *Anxa6* ^*−/−*^ mice for glucose production and secretion when pyruvate and glycerol were provided as substrate (Figure 3D‐E, AUC in Figure S4). In striking contrast, *Anxa6* ^*−/−*^ mice showed a significantly reduced production and secretion of glucose 60 min after intraperitoneal injection of glutamine (Figure 3F, AUC in Figure S4). The lower glucose production and secretion in ANXA6‐deficient animals was more prominent when lactate was provided as substrate (Figure 3G, AUC in Figure S4). Strikingly, *Anxa6* ^*−/−*^ mice were unable to synthesized glucose when supplemented with alanine (Figure 3H, AUC in Figure S4), suggesting a hindrance in the glucose‐alanine cycle (Cahill cycle).

Taken together, these results demonstrate the limited capacity of *Anxa6* ^*−/−*^ mice to produce and secrete glucose from glutamine and lactate, and their complete inability to use alanine as a substrate for hepatic GNG in vivo.

### 3.4 ANXA6 Deficiency Do Not Affect Alanine Metabolization Capability of the Liver

**Figure 4:**
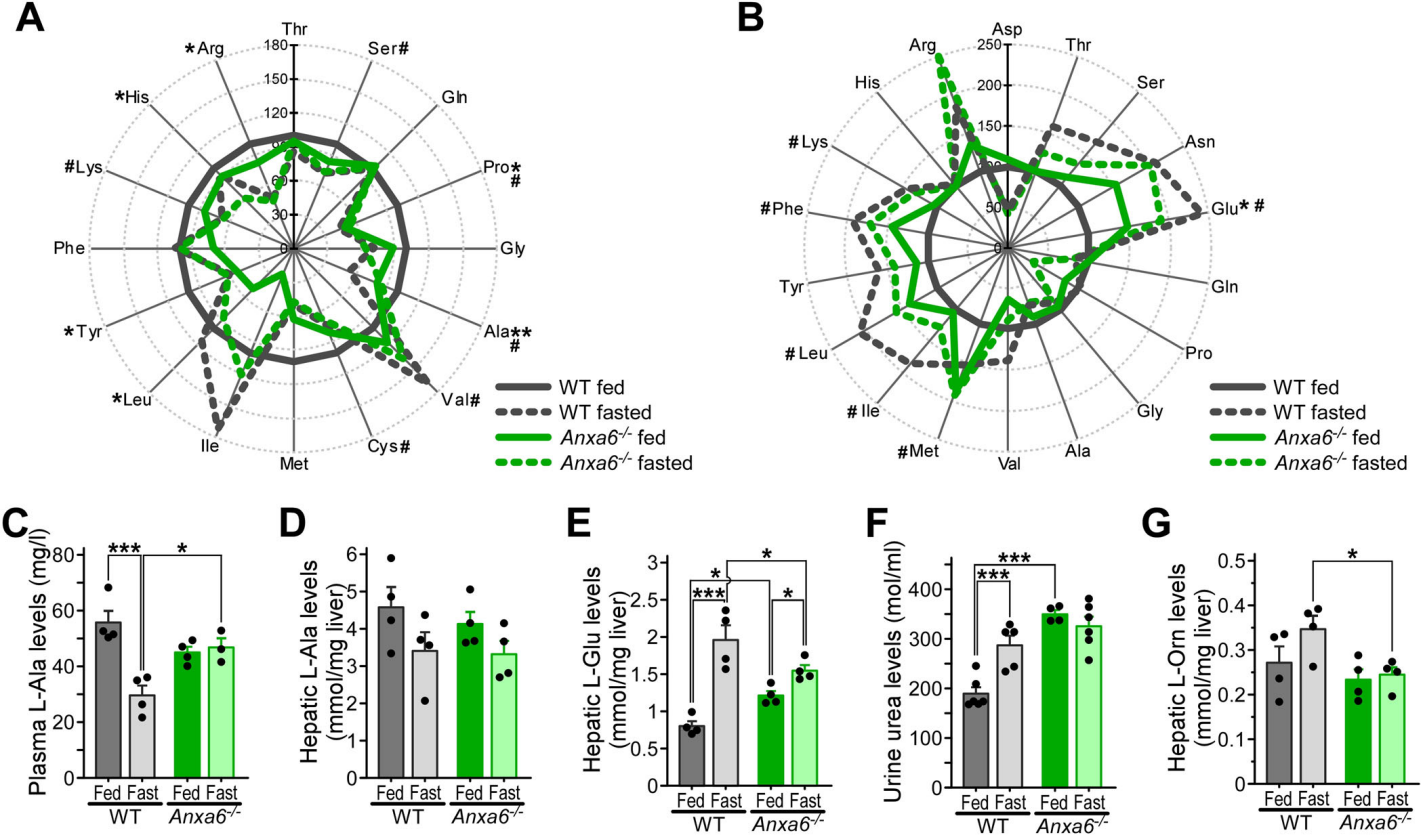
ANXA6 deficiency do not affect alanine metabolization capability of the mice liver. (A) Spider diagram representation of relative plasma threonine (l‐Thr), serine (l‐Ser), glutamine (l‐Gln), proline (l‐Pro), glycine (l‐Gly), alanine (l‐Ala), valine (l‐Val), cysteine (l‐Cys), methionine (l‐Met), isoleucine (l‐Ile), leucine (l‐Leu), tyrosine (l‐Tyr), phenylalanine (l‐Phe), lysine (l‐Lys), histidine (l‐His) and arginine (l‐Arg) levels of WT and *Anxa6* ^*−/−*^ mice fed and fasted for 24 h (n = 4 mice per group). (B) Spider diagram representation of relative hepatic aspartic acid (l‐Asp), threonine (l‐Thr), serine (l‐Ser), asparagine (l‐Asn), glutamic acid (l‐Glu), glutamine (l‐Gln), proline (l‐Pro), glycine (l‐Gly), alanine (l‐Ala), valine (l‐Val), methionine (l‐Met), isoleucine (l‐Ile), leucine (l‐Leu), tyrosine (l‐Tyr), phenylalanine (l‐Phe), lysine (l‐Lys), histidine (l‐His) and arginine (l‐Arg) levels of WT and *Anxa6* ^*−/−*^ mice fed and fasted for 24 h (n = 4 mice per group). (C) Plasma alanine levels in WT and *Anxa6* ^*−/−*^ mice fed and fasted for 24 h (n = 4 each group). (D) Hepatic alanine levels in WT and *Anxa6* ^*−/−*^ mice fed and fasted for 24 h (n = 4 each group). (E) Hepatic glutamic acid levels in WT and *Anxa6* ^*−/−*^ mice fed and fasted for 24 h (n = 4 each group). (F) Urine urea levels in WT and *Anxa6* ^*−/−*^ mice fed and fasted for 24 h (n = 4‐6 each group). (G) Hepatic ornithine levels in WT and *Anxa6* ^*−/−*^ mice fed and fasted for 24 h (n = 4 each group). Data are expressed as means ± SEM. Data was analysed by two‐way ANOVA with Bonferroni's post‐hoc test, *p < 0.05, **p < 0.01, ***p < 0.001 comparing *Anxa6* ^*−/−*^ to WT mice. In panels (A) and (B), # indicates lack of expected differences between fed and fast state in *Anxa6* ^*−/−*^ compared to WT mice.

Based on the data shown above, the failure of *Anxa6* ^*−/−*^ mice to maintain blood glucose levels during fasting was not due to a dysfunction of the glucose and insulin regulatory axis, but the inability to produce glucose mainly from alanine, the key gluconeogenic substrate during fasting. To substantiate these findings, we next compared amino acid levels in plasma (Figure 4A) and liver extracts (Figure 4B) from fed and 24 h fasted WT and *Anxa6* ^*−/−*^ mice. Amino acid levels are presented relative to WT‐fed controls, and the complete amino acid profile is provided in Supplementary Tables S1 and S2. The hash symbol (#) indicates cases where the expected fasting‐induced changes observed in WT mice were absent in *Anxa6* ^*−/−*^ mice. In *Anxa6* ^*−/−*^ mice, plasma alanine levels remained unchanged after fasting, suggesting an impaired ability to utilize alanine as a gluconeogenic substrate or a reduction in muscle‐derived alanine release (Figure 4C). In contrast, WT mice showed the expected significant decrease in plasma alanine upon fasting, consistent with its normal mobilization and use in gluconeogenesis. Furthermore, liver alanine levels were comparable in both strains of mice (Figure 4D). Yet, elevation of liver glutamate levels after fasting was much more prominent in WT compared to *Anxa6* ^*−/−*^ animals (Figure 4E), indicating a higher metabolic flux from alanine to pyruvate in WT mice. *Anxa6* ^*−/−*^ mice exhibited elevated urinary urea levels in both fed and fasted states (Figure 4F), suggesting increased amino acid deamination even under fed conditions. However, hepatic levels of ornithine (Figure 4G), urea, and branched‐chain amino acid (BCAAs) (Figure S5) remained low in fasted *Anxa6* ^*−/−*^ mice, indicating a compromised hepatic capacity for amino acid catabolism during fasting. Thus, based on these analyses, impaired ability to drive hepatic GNG via alanine appears a likely factor for the development of hypoglycaemia in fasted *Anxa6* ^*−/−*^ mice.

**Figure 5:**
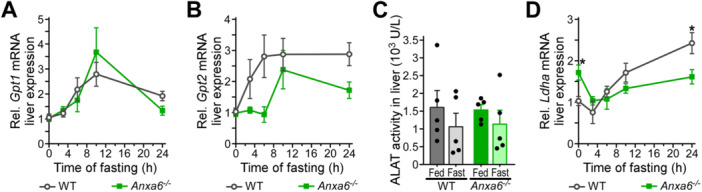
Hepatic alanine metabolization capacity in *Anxa6* ^*−/−*^ mice. (A) Relative liver mRNA expression levels of alanine aminotransferase 1 (*Gpt1*) during fasting in WT and *Anxa6* ^*−/−*^ mice liver (n = 5 per group and time point). (B) Relative liver mRNA expression levels of alanine aminotransferase 2 (*Gpt2*) during fasting in WT and *Anxa6* ^*−/−*^ mice liver (n = 5 per group and time point). (C) Alanine aminotransferase activity in WT and *Anxa6* ^*−/−*^ liver during fasting (n = 5 per group and time point). (D) Relative liver mRNA expression levels of lactate dehydrogenase (*Ldha2*) during fasting in WT and *Anxa6* ^*−/−*^ mice liver (n = 5 per group and time point). Data are expressed as means ± SEM. Data was analysed by two‐way ANOVA with Bonferroni's post‐hoc test, *p < 0.05, **p < 0.01, ***p < 0.001 comparing *Anxa6* ^*−/−*^ to WT mice.

To further dissect and identify de‐regulated mechanisms of the alanine‐glucose cycle in ANXA6‐deficient hepatocytes, we analysed the expression of hepatic enzymes converting alanine into pyruvate: alanine aminotransferase (ALAT) 1 and 2 (*Gpt1/2*). In response to fasting, reduced *Gpt2* levels were observed in *Anxa6* ^*−/−*^ livers (Figure 5A‐B), yet ALAT activity was similar in liver extracts from WT and *Anxa6* ^*−/−*^ mice (Figure 5C), indicating that reduced *Gpt2* mRNA levels did not significantly impact on enzyme activity. In addition, hepatic lactate dehydrogenase (*Ldha*) was slightly reduced in *Anxa6* ^*−/−*^ mice after 24 h fasting (Figure 5D). Hence, at least at the mRNA level, hepatic expression of enzymes metabolizing alanine into pyruvate (Figure 5A‐C) and lactate into pyruvate (Figure 5D) were slightly reduced in the absence of ANXA6 after prolonged fasting, although their enzyme activity nor those enzymes converting pyruvate into glucose (Figure 3) appeared significantly affected.

### 3.5 Hepatic Alanine Transporters During Fasting in *Anxa6* ^*−/−*^ Mice

Similar to the lack of blood alanine clearance upon fasting in *Anxa6* ^*−/−*^ mice (Figure 4C), we previously demonstrated *Anxa6* ^*−/−*^ mice unable to use blood alanine for GNG after partial hepatectomy. This correlated with a failure to internalize alanine in *Anxa6* ^*−/−*^ hepatocytes (Alvarez‐Guaita et al. 2020), pointing at compromised hepatic uptake of alanine as a potentially underlying mechanism for the impaired alanine‐promoted GNG of fasted *Anxa6* ^*−/−*^ mice.

**Figure 6:**
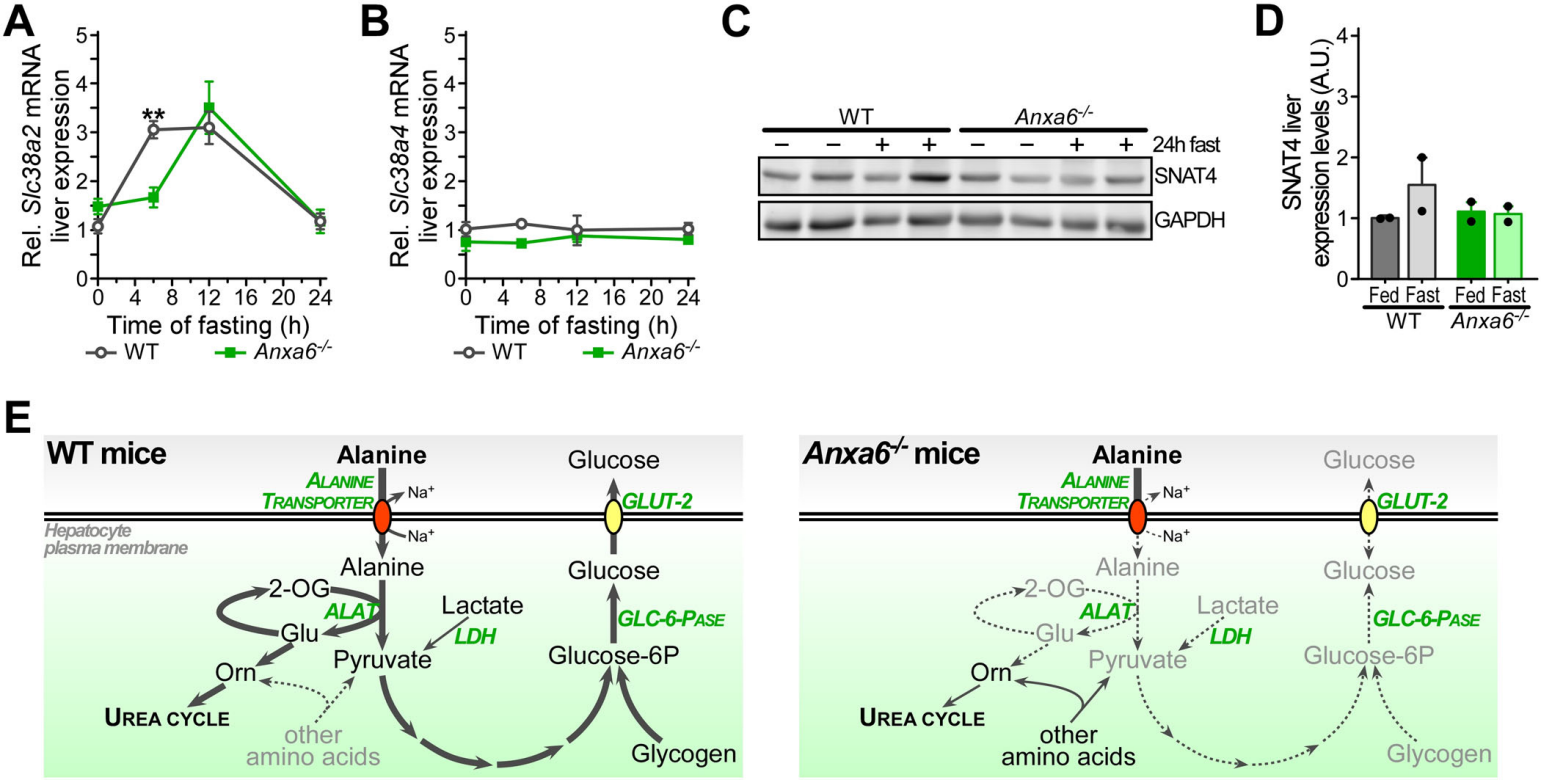
Hepatic alanine transporters during fasting in *Anxa6* ^*−/−*^ mice. (A) Relative liver mRNA expression levels of SNAT2 (*Slc38a2*) after 24 h fasting in WT and *Anxa6* ^*−/−*^ mice liver (n = 5 per group and time point). (B) Relative liver mRNA expression levels of SNAT4 (*Slc38a4*) after 24 h fasting in WT and *Anxa6* ^*−/−*^ mice liver (n = 5 per group and time point). (C) Relative expression of SNAT4 amino acid transporter after 24 h fasting in WT and *Anxa6* ^*−/−*^ mice liver (n = 2 per group and time point). (D) Relative quantification of SNAT4 amino acid transporter expression after 24 h fasting in WT and *Anxa6* ^*−/−*^ mice liver (n = 4 mice per group and time point). (E) Schematic representation of gluconeogenic pathway in WT and *Anxa6* ^*−/−*^ hepatocytes during fasting in mice. The lack of ANXA6 reduces hepatic alanine uptake and compromises alanine‐dependent gluconeogenesis required for blood glucose homeostasis during fasting. Abbreviations: 2‐OG, 2‐oxoglutarate; Glu, glutamic acid; Orn, ornithine; ALAT, alanine aminotransferase; LDH, lactate dehydrogenase; GLC‐6‐Pase, glucose‐6‐phosphatase. Data are expressed as means ± SEM. Data was analysed by two‐way ANOVA with Bonferroni's post‐hoc test, *p < 0.05, **p < 0.01, ***p < 0.001 comparing *Anxa6* ^*−/−*^ to WT mice.

We therefore first analysed mRNA levels of SNAT2 and 4 (*Slc38a2* and *Slc38a4*) and protein levels of SNAT4 (Figure 6A‐D). Elevation of hepatic SNAT2 mRNA levels was delayed in *Anxa6* ^*−/−*^ mice during fasting, while SNAT4 mRNA expression remained constant over the fasting period (Figure 6A‐B). Furthermore, elevation of SNAT4 protein levels after 24 h in WT mice was not observed in ANXA6‐deficient animals (Figure 6C, quantification in 6D). Together, these findings may indicate reduced amounts of SNATs being available for alanine uptake in fasted *Anxa6* ^*−/−*^ livers.

Altogether, *Anxa6* ^*−/−*^ mice showed an energetic imbalance during fasting that induced a fast drop in blood glucose levels and a faster switch from glycolytic to lipolytic metabolism without any indication of the development of a diabetic phenotype. In *Anxa6* ^*−/−*^ mice, the inability to maintain glycolytic metabolism in the initial stages of fasting was due to an impairment in alanine‐dependent GNG.

## 4 Discussion

The objective of this study was to examine the function of ANXA6 in maintaining glucose homeostasis under physiological conditions that reflected the fed and fasted states. *Anxa6* ^*−/−*^ mice exhibited glucose absorption and insulin response that were comparable to WT mice during the absorptive state. These findings were anticipated following the previous analysis of glucose metabolism after partial hepatectomy or upon a prolonged feeding of a high‐fat diet (Alvarez‐Guaita et al. 2020; Cairns et al. 2018). However, our findings indicate that ANXA6 plays a pivotal role in maintaining blood glucose levels during fasting, despite the apparent normal glucose metabolism observed under feeding conditions. The metabolic response analysis over a 24 h period using indirect calorimetry demonstrated that both WT and *Anxa6* ^*−/−*^ mice initiated lipid oxidation‐dependent energy production during the initial hours of the quiescent dark period. Though, the *Anxa6* ^*−/−*^ mice were unable to induce the lipid‐to‐carbohydrate catabolic switch at the end of the dark period in comparison to their control littermates. This was probably due to the inability of the *Anxa6* ^*−/−*^ mice to activate the hepatic gluconeogenic pathway, as observed during liver regeneration following partial hepatectomy (Alvarez‐Guaita et al. 2020). In accordance, the insulin‐sensitive control of systemic glucose levels, glucose absorption and secretion were not negatively affected in *Anxa6* ^*−/−*^ mice during regular feeding conditions.

Interestingly, our data demonstrated a notable decrease in blood glucose levels during the first stages of fasting in *Anxa6* ^*−/−*^ mice, in comparison to WT mice. This initially suggested an impairment of hepatic GNG or glycogen mobilization. However, the analysis of the expression of gluconeogenic key regulatory genes (*G6pc2*, *Pck1,* and *Fbp1*) revealed elevated levels of PEPCK expression in the liver during the basal fed state in *Anxa6* ^*−/−*^ mice, indicating its predisposition to GNG activation even during the fed state. A comparable phenotype has been observed in the liver glycogen synthase knockout (*Gys2* ^*−/−*^) mice, which exhibited elevated hepatic *Pck1* gene expression and PEPCK activity during the fed state (Irimia et al. 2010). Nevertheless, *Gys2* ^*−/−*^ mice displayed a 95% reduction in liver glycogen content during the fed state and exhibited insulin resistance (Irimia et al. 2010; Irimia et al. 2017), which was not observed in *Anxa6* ^*−/−*^ mice. The robust fasting‐induced hypoglycaemia observed in *Anxa6* ^*−/−*^ mice persisted despite the efficient mobilisation of elevated hepatic glycogen reserves at elevated degradation rates from the onset of the fasting state in these animals. This phenomenon correlated with a rapid decline in blood insulin levels and a rapid increase in blood glucagon levels. Taken together, these data suggest a very sensitive and elevated reaction capacity of *Anxa6* ^*−/−*^ hepatocytes facing a carbohydrate‐dependent energetic deficit, inducing a fast switch to a lipid‐catabolic metabolism.

The pronounced hypoglycaemia during the fasting state observed in *Anxa6* ^*−/−*^ mice correlated with its inability to sustain glucose production via GNG, particularly from alanine, the major gluconeogenic substrate in fasted WT mice (Wang et al. 2020). The pyruvate and glycerol tolerance tests indicated that there was no significant difference in blood glucose secretion between WT and *Anxa6* ^*−/−*^ mice. These results suggest that the inability to produce *de novo* glucose observed in *Anxa6* ^*−/−*^ mice was not due to a biochemical defect, but rather a limitation in the availability of GNG substrates during fasting, potentially leading to early metabolic exhaustion. In accordance, the urea cycle, which is essential for the detoxification of ammonium ions released during amino acid catabolism (Holeček 2024), appeared fully functional in *Anxa6* ^*−/−*^ mice as indicated by high urine urea levels during both fed and fasting states. Notably, data suggested that the urea cycle was continuously activated in *Anxa6* ^*−/−*^ mice due to a defective amino acid metabolism and/or turnover, which correlated with high urine production and water intake of *Anxa6* ^*−/−*^ mice. *Anxa6* ^*−/−*^ mice also showed lower glucose production via GNG from lactate but not pyruvate, which may relate to the lower *Ldha* expression levels observed in these *Anxa6* ^*−/−*^ mice during fasting.

The availability of gluconeogenic substrates in the liver is a critical determinant of the GNG flux rates (Rui 2014). Alanine availability for the liver is not affected in *Anxa6* ^*−/−*^ compared to WT mice, which showed lower but not significantly reduced levels of blood alanine during the fed state. As anticipated, 24 h fasting resulted in a reduction of blood alanine levels in WT mice, indicating the presence of a robust GNG‐dependent alanine uptake mechanism. The slight reduction in hepatic alanine levels and the increase in urine urea levels were also found to correlate with this alanine‐dependent GNG in WT mice. Interestingly, *Anxa6* ^*−/−*^ mice exhibited comparable blood alanine levels during both fed and fasting states, despite the absence of discernible alterations in hepatic ALAT activity. These findings demonstrate that alanine metabolic capacity, including enzyme activity, remains intact in *Anxa6* ^*−/−*^ mice. However, alanine uptake into hepatocytes is impaired during fasting, consistent with our previous observations in *Anxa6* ^*−/−*^ mice during liver regeneration (Alvarez‐Guaita et al. 2020) (Figure 6E).

Earlier research in *Anxa6* ^*−/−*^ mice demonstrated that SNAT4 recycling to the sinusoidal plasma membrane after partial hepatectomy in *Anxa6* ^*−/−*^ mice was impaired, which correlated with compromised alanine uptake and *de novo* glucose production (Alvarez‐Guaita et al. 2020). Concurrently, Curnock and colleagues demonstrated that the nutrient‐sensing and adaptive delivery of SNAT2 to the plasma membrane depends on the retromer complex, which prevents SNAT2 degradation in lysosomes (Curnock et al. 2019). On the other hand, the promoter of the retromer complex subunits *VPS35*, *VPS26A* and *SNX27* genes contain TFEB/TFE3‐responsive elements, which regulate its expression in response to amino acid starvation or selective glutamine depletion (Curnock et al. 2019). Then, the nutrient‐dependent expression of retromer proteins has been shown to modulate the retromer‐mediated retrieval and recycling of amino acid transporter proteins. Intriguingly, SNAT2 gene expression is upregulated during fasting, suggesting a further level of nutrient‐dependent regulation of this amino acid transporter. The recruitment and assembly of retromer components requires active RAB7 (RAB7‐GTP) (Rojas et al. 2008). In contrast, upregulation of the RAB7‐GAP TBC1D5 inhibits retromer complex formation through downregulating RAB7 activity (Seaman et al. 2009). Interestingly, ANXA6 has been demonstrated to directly interact with the RAB7‐GAP TBC1D15 to regulate RAB7‐GTP levels, with consequences for intracellular cholesterol trafficking (Meneses‐Salas et al. 2020). These studies may point at a yet unknown link between ANXA6 and RAB7 for the regulation of SNAT localization in a nutrient‐sensing manner.

Altogether, our findings identify that ANXA6 deficiency causes an inability to maintain glycolytic metabolism under fasting conditions due to impaired alanine‐dependent GNG.

## Author Contributions

David Sebastian, Antonio Zorzano, Francesc Tebar, Carlos Enrich, Thomas Grewal and Carles Rentero designed the experiments. Anna Alvarez‐Guaita, Marc Bernaus‐Esqué, Patricia Blanco‐Muñoz, Yangjing Liu, David Sebastian, Elsa Meneses‐Salas, Mai K Linh Nguyen, and Carles Rentero performed and analysed the experiments. Anna Alvarez‐Guaita, Marc Bernaus‐Esqué, Patricia Blanco‐Muñoz, Yangjing Liu, David Sebastian and Carles Rentero prepared figures. Antonio Zorzano, Francesc Tebar, Carlos Enrich, Thomas Grewal and Carles Rentero drafted the manuscript. Anna Alvarez‐Guaita, Marc Bernaus‐Esqué, Patricia Blanco‐Muñoz, Yangjing Liu, David Sebastian, Elsa Meneses‐Salas, Mai K Linh Nguyen, Antonio Zorzano, Francesc Tebar, Carlos Enrich, Thomas Grewal and Carles Rentero edited, revised, and approved the final version of the manuscript.

## Conflicts of Interest

The authors declare no conflicts of interest.

## Supporting information

AnxA6‐Glc_paper_JCP‐SupplMat_R2.

## Data Availability

The data that support the findings of this study are available from the corresponding author upon reasonable request.
