# Supplementary material for: Fasting‐Induced Hepatic Gluconeogenesis Is Compromised In Anxa6 −/− Mice
Source: J Cell Physiol. 2025 Aug 13;240(8):e70084. doi: 10.1002/jcp.70084 (PMC12349248; doi:10.1002/jcp.70084)
Supplement: Supplementary file 1 — AnxA6‐Glc_paper_JCP‐SupplMat_R2. [file JCP-240-0-s001.docx]

**SUPPLEMENTARY DATA**

**Fasting-induced hepatic gluconeogenesis is compromised in *Anxa6^-/-^* mice**

Anna Alvarez-Guaita; Marc Bernaus-Esqué; Patricia Blanco-Muñoz; Yangjing Liu; David Sebastian; Elsa Meneses-Salas; Mai K Linh Nguyen; Antonio Zorzano; Francesc Tebar; Carlos Enrich; Thomas Grewal; Carles Rentero

**Supplementary Figures:**

| **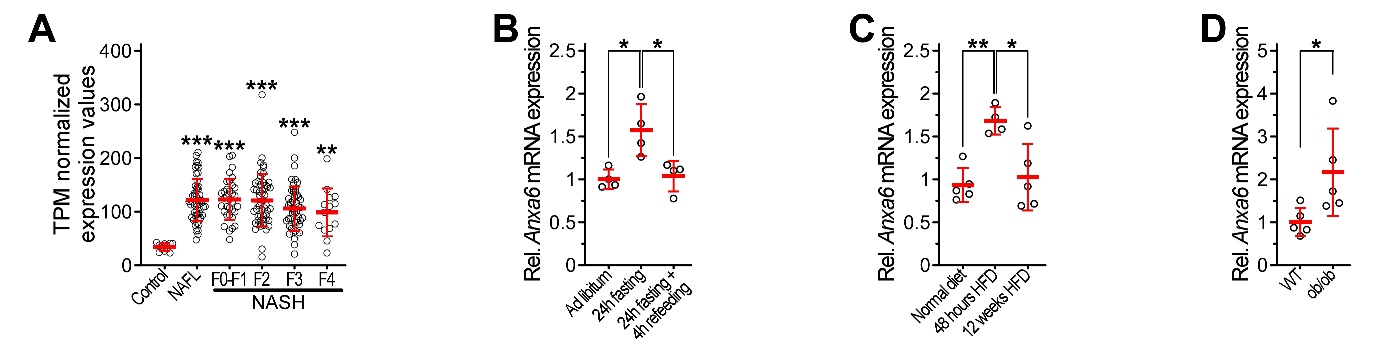** |
| --- |
| **Figure S1**. (A) Hepatic expression levels of *Anxa6* mRNA on a cohort of 206 individuals (381 NAFLD and 22 control samples) representing the full histological range from normal liver tissue to NASH-cirrhosis (Govaere et al., 2020). All samples were grouped according to histopathological disease grade and stage: NAFL and NASH with different fibrosis stages F0, F1, F2, F3, and F4 in the original study. (B) Hepatic expression levels of *Anxa6* mRNA in mice fed *ad libitum*, subject to a 24 hour fast or a 24 hour fast followed by a 4 hour refeeding (n=4 each group). (C) Hepatic expression levels of *Anxa6* mRNA in mice fed with normal diet or high-fat diet (HFD) for 48 hours or 12 weeks (n=5 each group). (D) Hepatic expression levels of *Anxa6* mRNA in WT or *ob/ob* mice (n=5 each group). Data obtained from publicly available microarray dataset (GSE135251 for panel A (Govaere et al., 2020); and GSE85439 for panels B, C and D (Yang et al., 2016)) deposited in NCBI Gene Expression Omnibus (GEO) repository and analysed with GEO2R (Barret et al., 2013). Data are expressed as means ± SEM. Data was analysed by one-way ANOVA with Bonferroni’s post-hoc test (panels A, B and C) or unpaired *t* test (panel D), *P<0.05, **P<0.01. |

| 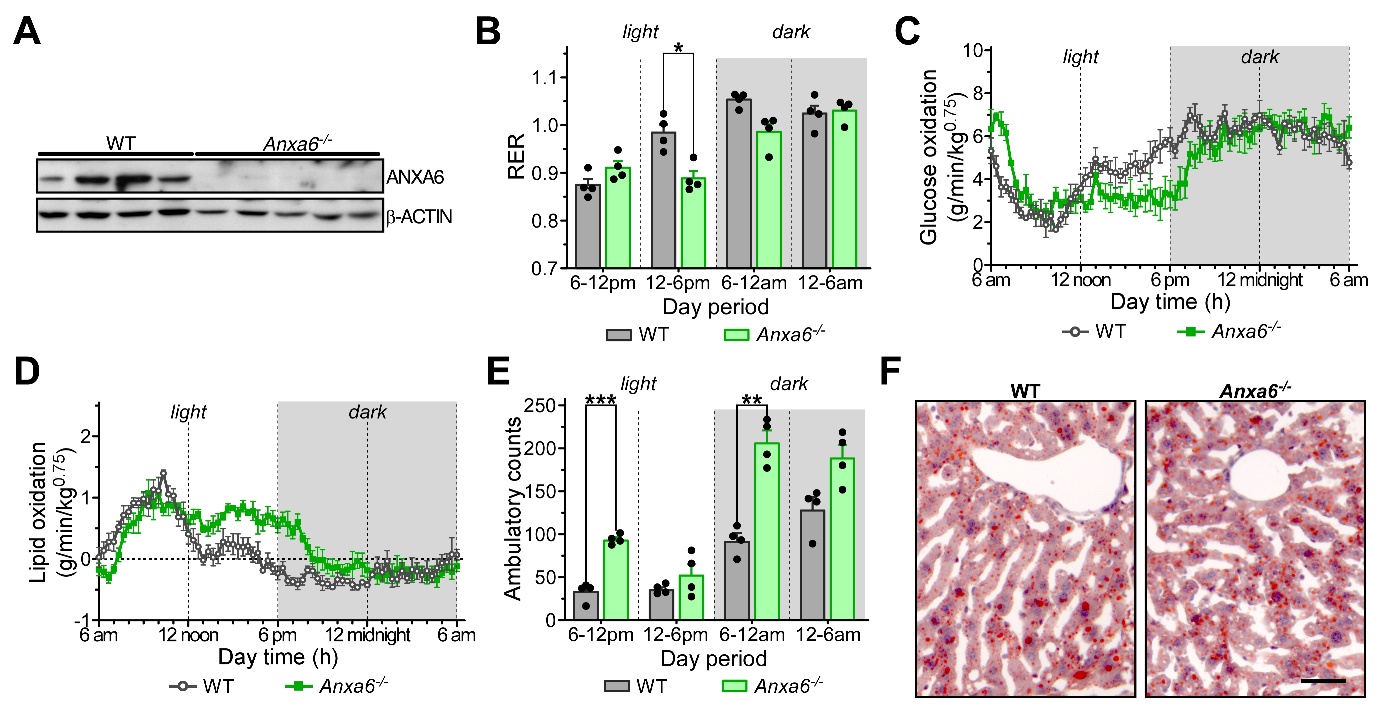 |
| --- |
| **Figure S2**. (A) Relative expression of ANXA6 in WT and *Anxa6^-/-^* mice liver (n= 4-5 per group). (B) Respiratory exchange ratio (RER) expressed as the mean of 6-hour period during day and night-time of WT and *Anxa6^-/-^* mice (n=4 mice per group). (C) Glucose oxidation from WT and *Anxa6^-/-^* mice measured every 20 minutes (n=4 mice per group). (D) Lipid oxidation from WT and *Anxa6^-/-^* mice measured every 20 minutes (n=4 mice per group). (E) Mice ambulation expressed as the mean of 6-hour period during day and night-time of WT and *Anxa6^-/-^* mice (n=4 mice per group). (F) Representative images of liver sections stained with Oil Red O from WT and *Anxa6^-/-^* mice fasted for 18 hours. Data are expressed as means ± SEM. Data was analysed by two-way ANOVA with Bonferroni’s post-hoc test, *P<0.05, **P<0.01, ***P<0.001 comparing *Anxa6^-/-^* to WT mice. Scale bar, 50 µm. |

| 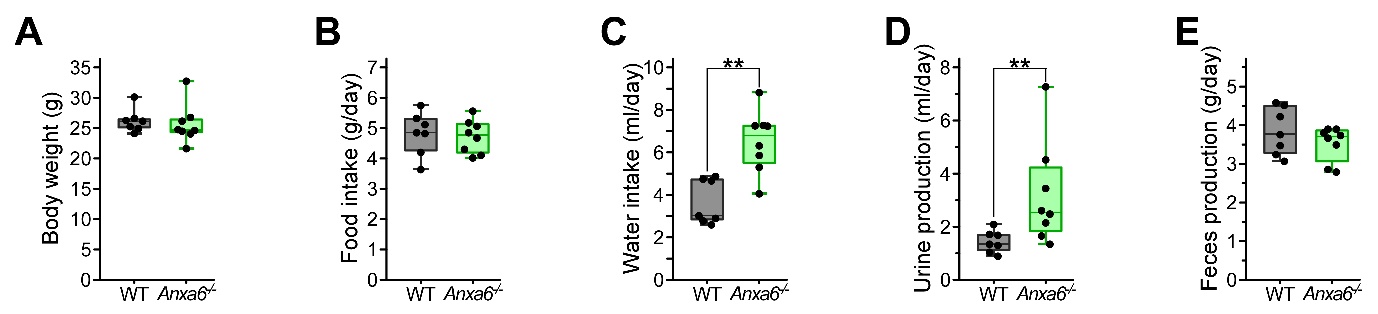 |
| --- |
| **Figure S3**. (A) Body weight of 10-week-old WT and *Anxa6^-/-^* mice (n=7 mice per group). (B) Food intake of WT and *Anxa6^-/-^* mice (n=7 mice per group). (C) Water intake of WT and *Anxa6^-/-^* mice (n=7 mice per group). (D) Urine production of WT and *Anxa6^-/-^* mice (n=7 mice per group). (E) Faeces production of WT and *Anxa6^-/-^* mice (n=7 mice per group). Data are expressed as means ± SEM. Data was analysed by unpaired *t* test, *P<0.05, **P<0.01, ***P<0.001 comparing *Anxa6^-/-^* to WT mice. |

| 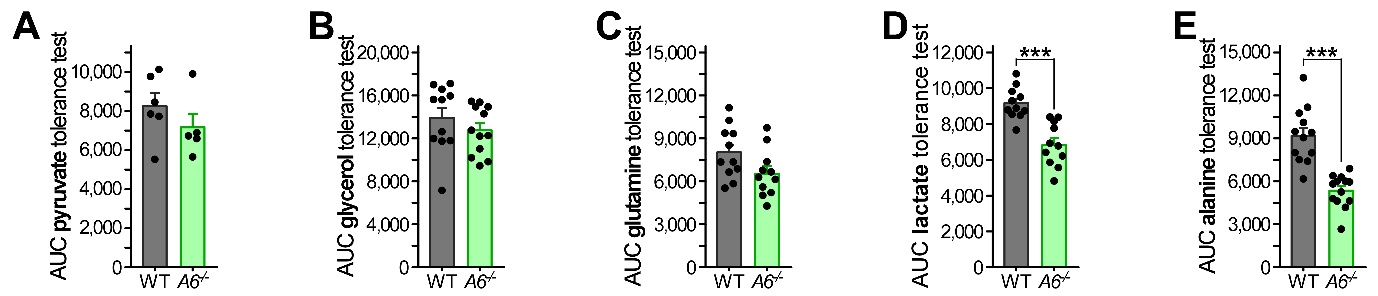 |
| --- |
| **Figure S4**. (A) AUC from pyruvate tolerance test of WT and *Anxa6^-/-^* mice (n=6 mice per group) after 24 hours fasting administrating i.p. 2 g/kg of pyruvate. (B) AUC from glycerol tolerance test of WT and *Anxa6^-/-^* mice (n=6 mice per group) after 24 hours fasting administrating i.p. 2 g/kg of glycerol. (C) AUC from glutamine tolerance test of WT and *Anxa6^-/-^* mice (n=11 mice per group) after 24 hours fasting administrating i.p. 2 g/kg of glutamine. (D) AUC from lactate tolerance test of WT and *Anxa6^-/-^* mice (n=11 mice per group) after 24 hours fasting administrating i.p. 2 g/kg of lactate. (E) AUC from alanine tolerance test of WT and *Anxa6^-/-^* mice (n=12 mice per group) after 24 hours fasting administrating i.p. 2 g/kg of alanine. Data are expressed as means ± SEM. Data was analysed by unpaired *t* test, *P<0.05, **P<0.01, ***P<0.001 comparing *Anxa6^-/-^* to WT mice. |

| **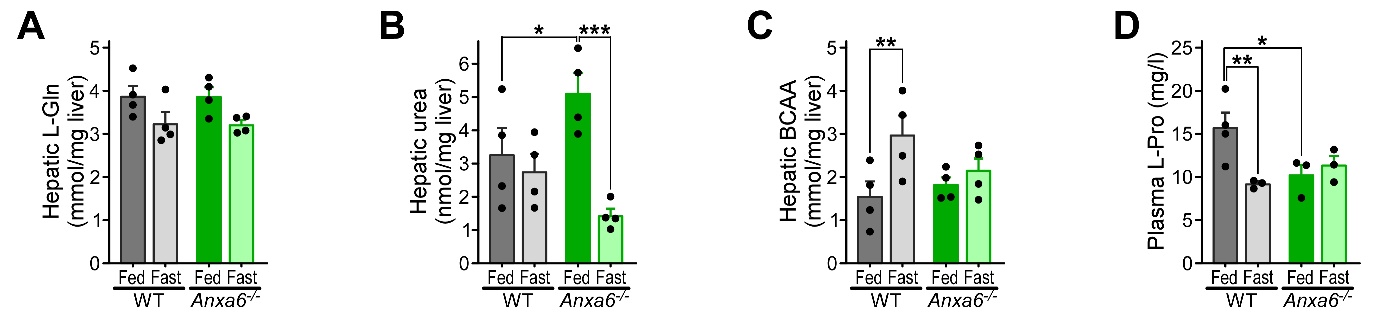** |
| --- |
| **Figure S5**. (A) Hepatic glutamine levels in WT and *Anxa6^-/-^* mice fed and fasted for 24 hours (n=4 each group). (B) Hepatic urea levels in WT and *Anxa6^-/-^* mice fed and fasted for 24 hours (n=4 each group). (C) Hepatic branched chain amino acid (BCAA) levels in WT and *Anxa6^-/-^* mice fed and fasted for 24 hours (n=4 each group). (D) Hepatic proline levels in WT and *Anxa6^-/-^* mice fed and fasted for 24 hours (n=4 each group). Data are expressed as means ± SEM. Data was analysed by two-way ANOVA with Bonferroni’s post-hoc test, *P<0.05, **P<0.01, ***P<0.001 comparing *Anxa6^-/-^* to WT mice or fasted to fed mice. |

**REFERENCES**

Barret, T., Wilhite, S.E., Ledoux, P., Evangelista, C., Kim, I.F., Tomashevsky, M., Marshall, K.A.,… Soboleva, A. (2013). NCBI GEO: archive for functional genomics data sets – update. *Nuc Acid Res*, *41*(D1), D991-D995. doi: 10.1093/nar/gks1193

Govaere, O., Cockell, S., Tiniakos, D., Queen, R., Younes, R., Vacca, M.,… Anstee, Q.M. (2020). Transcriptomic profiling across the nonalcoholic fatty liver disease spectrum reveals gene signatures for steatohepatitis and fibrosis. *Sci Transl Med*, *12*(572). doi: 10.1126/scitranslmed.aba4448

Yang, L., Li, P., Yang, W., Ruan, X., Keisewetter, K., Zhu, J., & Cao, H. (2016). Integrative Transcriptome Analyses of Metabolic Responses in Mice Define Pivotal LncRNA Metabolic Regulators. *Cell Metab*, *24*, 627-639. doi: 10.1016/j.cmet.2016.08.019

**Supplementary Tables:**

**Table S1.** Plasma amino acid levels (mg/l) of WT and *Anxa6^-/-^* mice fed and fasted for 24 hours.

|  | WT_1 | WT_2 | WT_3 | WT_4 | WT_1 | WT_2 | WT_3 | WT_4 | A6ko_1 | A6ko_2 | A6ko_3 | A6ko_4 | A6ko_1 | A6ko_2 | A6ko_3 | A6ko_4 |
| --- | --- | --- | --- | --- | --- | --- | --- | --- | --- | --- | --- | --- | --- | --- | --- | --- |
|  | fed | fed | fed | fed | fasted | fasted | fasted | fasted | fed | fed | fed | fed | fasted | fasted | fasted | fasted |
| Ala | 68.31 | 51.54 | 50.37 | 52.69 | 34.99 | 36.06 | 26.33 | 21.69 | 49.41 | 40.15 | 47.08 | 43.40 | 41.59 | 46.18 | n.a. | 52.85 |
| Thr | 33.06 | 25.28 | 25.40 | 27.79 | 21.24 | 27.90 | 23.35 | 22.93 | 24.36 | 19.30 | 24.63 | 37.65 | 31.61 | 30.23 | 15.15 | 29.01 |
| Ser | 21.95 | 16.56 | 16.47 | 16.62 | 12.59 | 15.83 | 10.97 | 11.81 | 15.39 | 13.63 | 13.09 | 17.55 | 14.50 | 15.87 | 7.41 | 14.10 |
| Gln | 66.00 | 64.81 | 91.85 | 83.07 | 61.86 | 76.65 | 86.85 | 78.78 | 84.26 | 76.02 | 94.42 | 111.64 | 66.39 | 78.45 | 36.07 | 109.05 |
| Pro | 16.03 | 20.21 | 15.00 | 11.20 | 9.56 | 8.65 | 9.29 | 0.00 | 11.62 | 7.58 | 11.35 | 0.00 | 9.39 | 13.17 | 0.00 | 11.42 |
| Gly | 29.07 | 26.91 | 23.96 | 18.44 | 18.14 | 18.74 | 18.03 | 15.80 | 22.99 | 17.49 | 19.05 | 27.27 | 17.18 | 17.64 | 9.80 | 17.81 |
| Val | 33.27 | 35.47 | 33.29 | 27.67 | 23.58 | 14.05 | 21.25 | 31.87 | 29.60 | 32.58 | 14.86 | 28.80 | 29.60 | 32.58 | 14.86 | 28.80 |
| Cys | 12.90 | 11.03 | 19.44 | 13.89 | 8.92 | 9.61 | 10.24 | 12.62 | 11.99 | 11.94 | 12.71 | 9.49 | 9.52 | 11.70 | 6.99 | 11.01 |
| Met | 15.17 | 14.06 | 16.71 | 9.95 | 4.72 | 8.61 | 8.71 | 6.00 | 13.07 | 11.03 | 10.85 | 0.00 | 12.00 | 9.39 | 0.00 | 4.79 |
| Ile | 14.99 | 0.00 | 5.69 | 10.85 | 12.87 | 14.25 | 11.39 | 16.14 | 0.00 | 0.00 | 7.85 | 0.00 | 10.43 | 12.95 | 5.84 | 9.05 |
| Leu | 35.01 | 24.56 | 19.63 | 20.44 | 31.38 | 32.47 | 24.94 | 25.57 | 17.33 | 16.00 | 16.25 | 0.00 | 25.67 | 26.04 | 10.11 | 24.16 |
| Tyr | 31.84 | 31.80 | 17.32 | 28.21 | 17.27 | 17.27 | 16.60 | 15.66 | 18.30 | 21.58 | 18.79 | 0.00 | 15.65 | 20.29 | 8.14 | 23.85 |
| Phe | 18.72 | 16.02 | 10.54 | 11.68 | 14.24 | 16.57 | 11.61 | 16.87 | 12.95 | 13.69 | 13.55 | 0.00 | 18.24 | 17.38 | 8.36 | 13.18 |
| Lys | 71.68 | 55.41 | 45.36 | 52.79 | 41.18 | 36.17 | 38.38 | 36.02 | 50.08 | 50.36 | 48.39 | 40.83 | 44.49 | 37.50 | 26.56 | 42.18 |
| Arg | 34.18 | 25.42 | 22.68 | 28.48 | 11.97 | 16.04 | 13.12 | 11.60 | 21.46 | 20.26 | 24.95 | 24.21 | 18.31 | 14.11 | 0.00 | 17.09 |
| His | 14.60 | 13.82 | 13.00 | 9.74 | 12.26 | 11.34 | 10.57 | 13.17 | 10.78 | 10.10 | 9.81 | 15.13 | 10.23 | 9.87 | 4.10 | 7.93 |

**Table S2**. Hepatic amino acid levels (µmol/g) of WT and *Anxa6^-/-^* mice fed and fasted for 24 hours.

|  | WT_1 | WT_2 | WT_3 | WT_4 | WT_1 | WT_2 | WT_3 | WT_4 | A6ko_1 | A6ko_2 | A6ko_3 | A6ko_4 | A6ko_1 | A6ko_2 | A6ko_3 | A6ko_4 |
| --- | --- | --- | --- | --- | --- | --- | --- | --- | --- | --- | --- | --- | --- | --- | --- | --- |
|  | fed | fed | fed | fed | fasted | fasted | fasted | fasted | fed | fed | fed | fed | fasted | fasted | fasted | fasted |
| Asp | 3527.76 | 5189.25 | 4960.31 | 5032.15 | 2236.70 | 2149.51 | 2360.62 | 1857.01 | 4856.23 | 5355.09 | 5352.55 | 4739.71 | 1676.29 | 2003.01 | 1748.42 | 2007.26 |
| Thr | 563.87 | 748.46 | 416.90 | 248.22 | 1042.81 | 1034.45 | 436.90 | 623.15 | 474.66 | 453.56 | 561.76 | 497.44 | 791.02 | 802.17 | 436.12 | 430.31 |
| Ser | 893.82 | 1064.66 | 763.57 | 405.55 | 1862.69 | 1690.24 | 620.62 | 1139.29 | 775.47 | 845.05 | 1024.76 | 907.43 | 1401.77 | 1340.47 | 758.04 | 727.93 |
| Asn | 270.44 | 208.57 | 475.66 | n.a. | 688.69 | 607.53 | 344.81 | 364.29 | 353.66 | 358.58 | 400.69 | 354.81 | 729.75 | 544.15 | 349.05 | 318.99 |
| Glu | 750.96 | 994.74 | 788.68 | 701.28 | 2223.24 | 2360.95 | 1571.32 | 1709.13 | 1133.33 | 1117.02 | 1388.58 | 1229.60 | 1436.97 | 1760.37 | 1541.48 | 1478.07 |
| Gln | 4524.48 | 3397.29 | 3672.56 | 3912.52 | 3144.65 | 4033.05 | 2852.71 | 2990.61 | 4307.45 | 4094.56 | 3787.35 | 3353.72 | 3029.46 | 3405.52 | 3080.76 | 3372.49 |
| Pro | 165.12 | 334.60 | 91.32 | 166.29 | 201.07 | 292.05 | 196.12 | 93.65 | 257.45 | n.a. | 190.37 | 168.58 | n.a. | n.a. | 95.27 | 179.05 |
| Gly | 2886.83 | 3563.15 | 3125.58 | 2204.13 | 2933.33 | 3056.76 | 2071.47 | 2428.04 | 2990.24 | 3171.55 | 2729.44 | 2416.93 | 2489.99 | 2718.23 | 2289.12 | 2307.12 |
| Ala | 3347.65 | 5902.22 | 4238.30 | 4869.84 | 4377.98 | 3570.43 | 3714.26 | 2081.09 | 5062.87 | 3640.59 | 4163.14 | 3686.48 | 2823.42 | 3668.90 | 2708.68 | 4158.52 |
| Val | 519.34 | 577.74 | 139.38 | 220.63 | 359.48 | 811.58 | 335.50 | 530.29 | 226.15 | n.a. | 372.22 | 329.60 | 331.22 | 418.06 | 243.06 | 293.86 |
| Met | 250.89 | 334.08 | 233.49 | n.a. | 690.83 | 619.11 | 279.54 | 371.69 | 273.31 | 247.00 | 340.44 | 301.46 | 517.53 | 469.90 | 297.48 | 357.96 |
| Ile | 242.76 | 199.76 | 126.51 | 125.75 | 388.99 | 424.55 | 215.50 | 274.74 | 136.04 | 137.52 | 239.34 | 211.94 | 235.35 | 308.86 | 159.94 | 193.02 |
| Leu | 1053.52 | 1614.93 | 973.80 | 388.91 | 2692.05 | 2768.90 | 1351.01 | 1692.20 | 1176.69 | 1381.17 | 1629.71 | 1443.12 | 1961.41 | 2004.68 | 1073.34 | 1356.15 |
| Tyr | 467.39 | 530.42 | 274.73 | 184.35 | 827.68 | 672.11 | 397.83 | 459.92 | 398.10 | 427.48 | 440.30 | 389.89 | 593.52 | 711.04 | 333.75 | 410.34 |
| Phe | 458.11 | 757.79 | 475.04 | 162.45 | 1271.10 | 1160.25 | 500.00 | 664.15 | 644.99 | 678.94 | 728.47 | 645.07 | 1002.36 | 953.68 | 549.69 | 676.54 |
| Lys | 930.93 | 838.09 | 970.70 | 684.92 | 1590.67 | 1345.05 | 1129.77 | 858.20 | 771.68 | 851.19 | 1057.50 | 936.42 | 1319.59 | 1338.80 | 1049.69 | 854.33 |
| His | 799.77 | 814.30 | 775.97 | 554.05 | 962.84 | 735.29 | 662.02 | 651.59 | 658.27 | 693.45 | 822.01 | 727.89 | 893.37 | 772.58 | 638.49 | 629.75 |
| Arg | 182.10 | 156.66 | 284.96 | n.a. | 571.87 | 271.69 | 164.03 | 144.44 | 175.20 | 279.22 | 203.99 | 180.63 | 640.21 | 506.36 | 228.86 | 181.70 |
